# Asymptomatic malaria and anaemia among pregnant women during high and low malaria transmission seasons in Burkina Faso: household-based cross-sectional surveys in Burkina Faso, 2013 and 2017

**DOI:** 10.1186/s12936-021-03703-4

**Published:** 2021-05-01

**Authors:** Toussaint Rouamba, Sékou Samadoulougou, Mady Ouédraogo, Hervé Hien, Halidou Tinto, Fati Kirakoya-Samadoulougou

**Affiliations:** 1 Clinical Research Unit of Nanoro, Institut de Recherche en Sciences de La Santé, Centre National de La Recherche Scientifique Et Technologique, 42, Avenue Kumda-Yoore, BP 218 Ouagadougou CMS 11, Ouagadougou, Burkina Faso; 2 Centre de Recherche en Epidémiologie, Biostatistique Et Recherche Clinique, Ecole de Santé Publique, Université Libre de Bruxelles (ULB), Route de Lennik, 808, 1070 Bruxelles, Belgium; 3 Evaluation Platform On Obesity Prevention, Quebec Heart and Lung Institute, Quebec, G1V 4G5 Canada; 4 Centre for Research On Planning and Development (CRAD), Laval University, Quebec, G1V 0A6 Canada; 5 Institut National de La Statistique Et de La Démographie [INSD], 01 BP 374, Ouagadougou 01, Ouagadougou, Burkina Faso; 6 Intitut National de Santé Publique (INSP), Centre Muraz, Bobo-Dioulasso, Burkina Faso; 7 Institut de Recherche en Sciences de La Santé, Centre National de La Recherche Scientifique Et Technologique, 42, Avenue Kumda-Yoore, BP 218 Ouagadougou CMS 11, Ouagadougou, Burkina Faso

**Keywords:** Asymptomatic carriage, Plasmodium, Haemoglobin, Community, Pregnant, Health district

## Abstract

**Background:**

Malaria in endemic countries is often asymptomatic during pregnancy, but it has substantial consequences for both the mother and her unborn baby. During pregnancy, anaemia is an important consequence of malaria infection. In Burkina Faso, the intensity of malaria varies according to the season, albeit the prevalence of malaria and anaemia as well as their risk factors, during high and low malaria transmission seasons is underexplored at the household level.

**Methods:**

Data of 1751 pregnant women from October 2013 to March 2014 and 1931 pregnant women from April 2017 to June 2017 were drawn from two cross-sectional household surveys conducted in 24 health districts of Burkina Faso. Pregnant women were tested for malaria in their household after consenting. Asymptomatic carriage was defined as a positive result from malaria rapid diagnostic tests in the absence of clinical symptoms of malaria. Anaemia was defined as haemoglobin level less than 11 g/dL in the first and third trimester and less than 10.5 g/dL in the second trimester of pregnancy.

**Results:**

Prevalence of asymptomatic malaria in pregnancy was estimated at 23.9% (95% CI 20.2–28.0) during the high transmission season (October–November) in 2013. During the low transmission season, it was 12.7% (95% CI 10.9–14.7) between December and March in 2013–2014 and halved (6.4%; 95% CI 5.3–7.6) between April and June 2017. Anaemia prevalence was estimated at 59.4% (95% CI 54.8–63.8) during the high transmission season in 2013. During the low transmission season, it was 50.6% (95% CI 47.7–53.4) between December and March 2013–2014 and 65.0% (95% CI 62.8–67.2) between April and June, 2017.

**Conclusion:**

This study revealed that the prevalence of malaria asymptomatic carriage and anaemia among pregnant women at the community level remain high throughout the year. Thus, more efforts are needed to increase prevention measures such as IPTp–SP coverage in order to reduce anaemia and contribute to preventing low birth weight and poor pregnancy outcomes.

## Background

Every year, about 25% of maternal deaths in hyperendemic malaria regions are due to malaria infection in pregnancy (MiP) [1]. In sub-Saharan African (SSA) countries, MiP is often asymptomatic, which is one of the main challenges in controlling MiP. Indeed, asymptomatic carriage has substantial health consequences for the mother, her unborn baby, and her future newborn. Adverse consequences of MiP for both the mother and her unborn baby include fetal loss [2–4], intrauterine growth retardation [5, 6], preterm delivery [5, 6], low birth weight [5–8], congenital malaria  [3, 9], perinatal mortality [2, 6], and increased risks of maternal anaemia [6, 7]. Several studies reported a high prevalence of asymptomatic MiP (ranging from 21 to 58.4%) in SSA countries [10–14], including Burkina Faso, and this represents a major public health problem since pregnant women living in these communities are not aware they are asymptomatic carriers of malaria parasites.

To protect women from asymptomatic MiP and its consequences, the World Health Organization (WHO) recommends combined intervention, including intermittent preventive treatment during pregnancy with sulfadoxine–pyrimethamine (IPTp–SP) and iron + folic acid supplementation during antenatal care (ANC) [15]. Albeit, the effectiveness of IPTp–SP seemed to be compromised by the spread of resistance to SP in most African countries [16, 17], several studies reported that the IPTp-SP continues to reduce the incidence of malaria parasites carriage and the malaria-attributable adverse pregnancy outcomes namely, low birthweight, and maternal anaemia [18–22]. Even in areas with excellent SP sensitivity, such as in many parts of West Africa, there is still a high prevalence of placental infections in women receiving less than three doses of SP, particularly during the peak of malaria transmission season [23].

In Burkina Faso, around three-quarters of the population use self-medication or traditional therapy when signs of malaria are suspected [24]. By implication, around one-quarter of the population attends a clinic when they feel ill or due to scheduled visits (namely ANC), and are treated when there is confirmed malaria. Consequently, asymptomatic MiP cases and anaemia remain untreated. The prevalence of asymptomatic MiP among women attending routine ANC was estimated at about 19–51% based on malaria rapid diagnostic tests (RDTs) [25–28], whereas anaemia prevalence was estimated at around 60% [27, 28]. There are only a few community-based published studies that have estimated the prevalence of MiP [29] at the household level; in this case, conducted in Nanoro and nested within a cluster-randomized controlled trial. In Burkina Faso, the majority of published data on the subject have been obtained in healthcare settings (i.e., in health facilities). It is, therefore, important to have better insight into asymptomatic MiP and its corollary anaemia, at the community level. This will provide useful indicators to guide decision-making regarding control strategies and to optimize interventions in the context of resource constraints.

This study, aimed to estimate the prevalence of asymptomatic carriage of malaria parasites and anaemia among pregnant women in their community through a household survey during low and high malaria transmission seasons in Burkina Faso. The study also aimed to identify the potential factors associated with asymptomatic carriage and anaemia among pregnant women surveyed at their household.

## Methods

### Study population, design and sampling procedures

This study analysed data collected from 24 health districts located in six regions of Burkina Faso (Boucle de Mouhoun, Center-East, Center-North, Center-West, North, and South-West). The population consisted of pregnant women who were present in the household during the survey visit and who consented to participate in the study. The main project, entitled “Impact Evaluation for Health Performance-Based Financing in Burkina Faso”, was designed to assess the impact of a performance-based financing strategy on the quality of healthcare delivery [30]. The latter aimed to strengthen the public sector and enhance general health, including health indicators, with a particular focus on maternal and child health. This impact assessment was performed through health facility- and household-based surveys and consisted of cross-sectional studies carried out in 2013/2014 (prior to project implementation) and 2017 (after the project implementation), in which the health districts were non-randomly selected. The study protocol, including the survey design, are published elsewhere [30]. Briefly, a two-stage stratified cluster sampling was carried-out where the household was the sampling unit. The first stage of the sampling consisted to randomly select a village within each health facility, and then create a sample frame that included all the households, which had at least one pregnant woman or a woman who delivered in the last two years. The second stage consisted to perform a systematic sampling of 15 households within each frame. In total 7844 and 8182 households were surveyed respectively in year 2013/2014 and 2017.

Data on sociodemographics, health behaviour, and economic factors as well as on the health conditions of pregnant women were collected for each selected household. These data were collected using a computer-assisted personal interview (CAPI) household questionnaire. From these two cross-sectional household surveys, data on 1751 and 1931 pregnant women were drawn in 2013/2014 (from October 2013 to March 2014) and 2017 (From April to June 2017). To provide a snapshot of the prevalence, the timeline of data collection in the field was for a full year of malaria transmission (i.e., for low and high transmission). The first data collection was conducted between October 2013 and March 2014, and the second one occurred from April to June 2017.

### Malaria diagnosis and estimation of haemoglobin

During the household-based surveys, blood samples were taken by finger prick to detect the presence of the malaria parasite and to measure haemoglobin level. In both surveys, malaria was confirmed by serology using RDT SD Bioline, which detects histidine-rich protein II (*HRP-II)*. HRP-II-based diagnostic test accuracy with microscopy as reference test for detection of malaria in asymptomatic pregnant women in Burkina Faso was 96.4% (95% CI 91–99) and 73.9 (95% CI 68–79), respectively, for sensitivity and specificity [31].

The haemoglobin level was measured by the haematological acid technique using a haemoglobinometer (HemoCue®). During the field surveys, quality controls were carried out on randomly selected malaria-positive samples (microscopy performed on thick blood smears). Similarly, quality controls were performed on haemoglobin measures using an automated haematology analyzer.

Since the definition of anaemia in pregnancy varies according to the trimester of pregnancy to reflect changes in blood volume, in this study the pregnant women was categorized as anemic if the haemoglobin level (Hb) was < 11 g/dL in the first trimester, < 10.5 g/dL in the second trimester and < 11 g/dL in the third trimester [32].

### Study variables

This study assessed two main binary outcomes: asymptomatic carriage of malaria parasites (Yes or No) and anaemia (Yes or No) according to the definition mentioned above [32]. The explanatory variables were based mainly on individual- and household-level characteristics. The selection of these explanatory variables was based on epidemiological interest as well as on previous studies that have shown the relationship between potential risk factors and MiP (or anaemia) [10, 25–27, 33, 34]. These explanatory variables for pregnant women were age (< 20, 20–30, and > 30 years old), parity [primigravida, secundigravida, and multigravida (≥ 3)], and gestational age [first trimester (≤ 3 months), and second (between 3–6 months) or third trimester (≥ 7 months)]. It is noticeable that during household surveys the gestational age was determined by recall of last menstrual cycle date. In addition, if the woman was already attended a health facility for her antenatal care, the enumerators checked from antenatal cards or other relevant documents.

Other variables included the mother’s education level (no education and educated), ownership of insecticide-treated nets in the household (No and ≥ 1), the household’s standard of living (very poor, poor, moderate rich, rich, and very rich), and place of residence (urban and rural). To analyze malaria transmission among pregnant women, the study period was cut into two seasons: a high transmission season from July to November and a low transmission season from December to June [35]. It is noticeable that in this study, haemoglobin level was not considered as predictor of malaria because, in this present study, anaemia was already considered as a main consequence of MiP.

### Statistical analysis

The prevalence of asymptomatic carriage was estimated based on the proportion of pregnant women who tested positive with the malaria RDT. Likewise, the prevalence of anaemia was determined as the proportion of pregnant women with a haemoglobin level less than 11 g/dL in the first and third trimester, and < 10.5 g/dL in the second trimester among those who underwent haematological testing. Descriptive statistics were used to document asymptomatic carriage of malaria parasites, anaemia, and sociodemographic characteristics of pregnant women. Cross-tabulations were performed separately for each time point of the survey.

For each main outcome (i.e., asymptomatic carriage and anaemia), both univariate and multivariable modified Poisson regressions with generalized estimating equations (GEE) were performed. The selection of variables for multivariable analysis was based on epidemiological interest and based on previous studies that exhibited links between potential risk factors and the study outcomes. The modified Poisson regression with GEE were used to estimate unadjusted and adjusted prevalence ratios (Adj. PRs) with 95% confidence intervals (CIs), and two-tailed p values < 0.05 were considered as statistically significant. The generalized variance-inflation factors were computed to check for multi collinearity between the explanatory variables [36].

All statistical analyses were performed with R statistical software (R Development Core Team, R Foundation for Statistical Computing, Vienna, Austria), and regression models were fitted using the “geepack” package.

## Results

### Population characteristics

A total of 7844 and 8182 households were included for the 2013/2014 and 2017 surveys, respectively. Of the households included in the main study, 21.8% (1709/7844) and 23.4% (1916/8182) had at least one pregnant woman for the 2013/2014 and 2017 surveys, respectively. The sociodemographic characteristics of the pregnant women for each survey are summarized in Table 1. The characteristics of the study population followed the same distribution, with the exception of age, parity, education, trimester of pregnancy, and malaria transmission season. In fact, about half of the women were between 20 and 30 years old, and most did not attend a formal school (97.4%). About 23.5% of pregnant women were survey during their first trimester of pregnancy. The majority of women lived in rural areas (92.3%) and were multigravidae (66.9%). Additionally, 100% of the women surveyed in 2017 were investigated during the low transmission season (between April and June). Regarding the 2013/2014 survey, 70.2% were investigated during the high transmission season (between October and November), whereas 29.8% were interviewed during the low transmission season (between December and mid-March). More than three-quarters of the women were investigated in the second or third trimester of pregnancy.

**Table 1:** Sociodemographic characteristics of pregnant women surveyed at the household level in Burkina Faso, 2013 and 2017

Characteristics	Year		p value
	2013–2014	2017	
Total (number)	1751	1931	
Age, n(%)			< **0.001**^a^
≥ 30	480 (27.4)	655 (33.9)	
20–30	883 (50.4)	982 (50.9)	
≤ 20	388 (22.2)	294 (15.2)	
Education, n(%)			< **0.001**^a^
No education	1737 (99.2)	1849 (95.8)	
Educated	14 (0.8)	82 (4.2)	
Household socioeconomic status, n(%)			**0.66**
Poorest	310 (17.7)	349 (18.1)	
Poor	371 (21.2)	374 (19.4)	
Middle quintile	358 (20.4)	388 (20.1)	
Rich	344 (19.6)	400 (20.7)	
Richest	368 (21.0)	420 (21.8)	
Place of residence, n(%)			**0.41**
Rural	1624 (92.7)	1776 (92.0)	
Urban	127 (7.3)	155 (8.0)	
Region, n(%)			**0.74**
Centre-North	412 (23.5)	443 (22.9)	
Boucle de Mouhoun	339 (19.4)	356 (18.4)	
Centre-East	218 (12.5)	270 (14.0)	
Centre-West	292 (16.7)	314 (16.3)	
North	380 (21.7)	416 (21.5)	
South-West	110 (6.3)	132 (6.8)	
Parity, n(%)			**< 0.001**^a^
Multigravida	1067 (60.9)	1396 (72.3)	
Secundigravida	364 (20.8)	325 (16.8)	
Primigravida	320 (18.3)	210 (10.9)	
Trimester of pregnancy, n(%)			**0.016**^a^
First	415 (23.7)	450 (23.3)	
Second	753 (43.0)	755 (39.1)	
Third	583 (33.3)	726 (37.6)	
Insecticide-treated nets, n(%)			**0.82**
At least one	1690 (96.5)	1860 (96.3)	
No insecticide-treated nets	61 (3.5)	71 (3.7)	

^a^significant at the 0.05 level

### Prevalence of asymptomatic carriage of malaria parasites among pregnant women surveyed at the household level

**Fig. 1:**
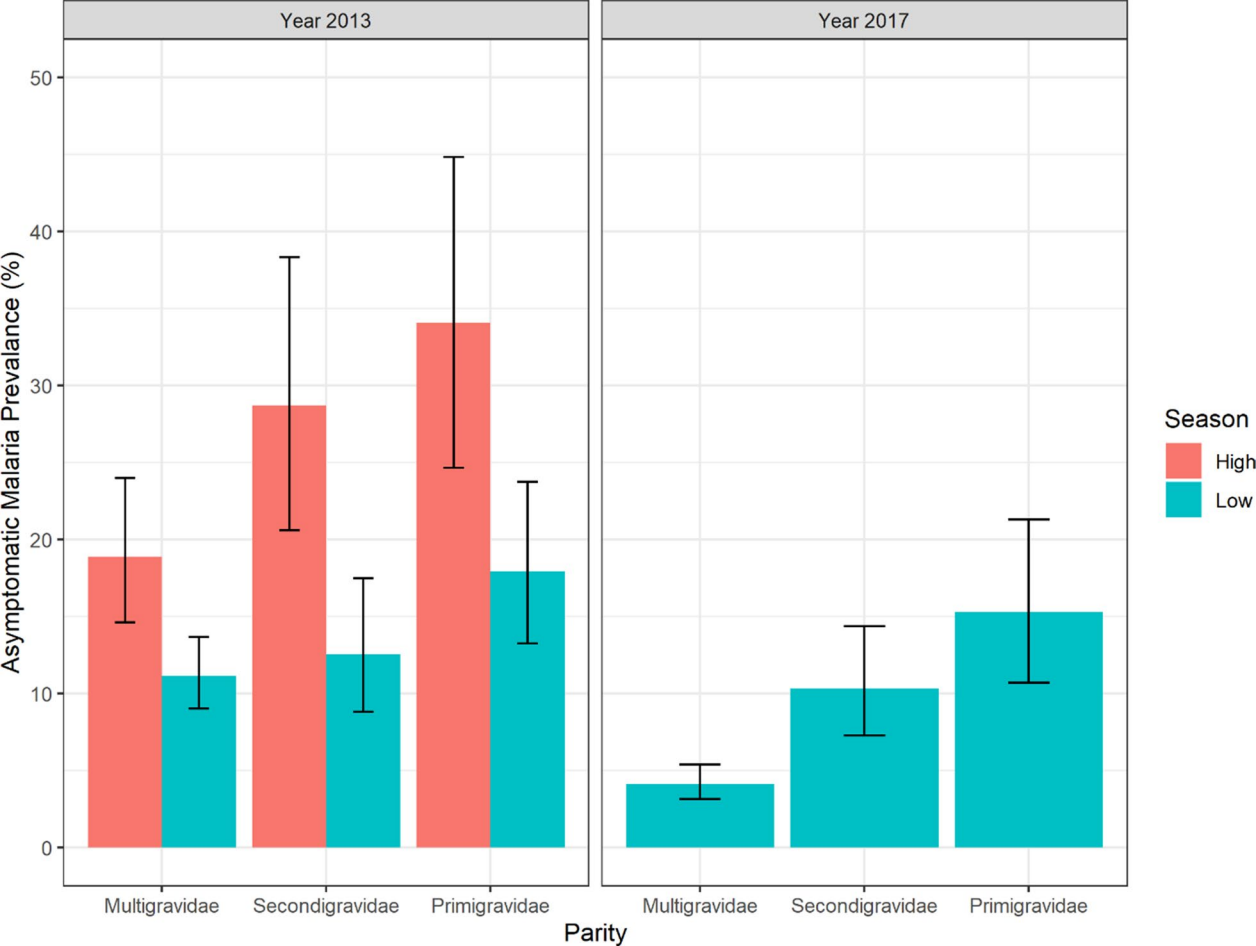
Prevalence of asymptomatic carriage of malaria parasites by gravidity according to the malaria transmission season. The high transmission season in 2013 covered the months between October and November. The low transmission season in 2013–2014 covered the months between December 2013 and March–2014. The low transmission season in 2017 covered the months between April and June 2017

The prevalence of asymptomatic carriage of malaria parasites and 95% CIs by gravidity and according to malaria transmission season is presented in the Fig. 1. The prevalence of asymptomatic carriage of the malaria parasites was estimated at 15.9% (95% CI 14.2–17.7) in 2013/2014. This prevalence was estimated at 12.7% (95% CI 10.9–14.7) for the low transmission season and 23.9% (95% CI 20.2–28.0) for the high transmission season. In 2017, between April and June (low transmission season), the prevalence of asymptomatic carriage of the malaria parasites was estimated at 6.4% (95% CI 5.3–7.6). The prevalence of asymptomatic-carriage was two-fold higher during the high season compared with low season. During the high season the prevalence of asymptomatic carriage of malaria parasites was estimated at 18.9% (95% CI 14.6–24.0), 28.7% (95% CI 20.6–38.3), and 34.1% (95% CI 24.7–44.8) respectively for multigravidae, secondigravidae and primigravidae.

**Table 2:** Risk factors associated with asymptomatic carriage of malaria parasites among pregnant women surveyed at the household level in Burkina Faso in 2013/2014

Potential factors	N	MiP cases	Univariate analysis		Multivariable analysis	
			PR (95% CI)	p	Adj. PR (95% CI)	p
Age				< **0.001**^a^		< **0.001**^a^
≥ 30	468	59	1		1	
20–30	380	122	1.13 (0.84–1.51)		1.07 (0.80–1.38)	
≤ 20	859	90	1.88 (1.39–2.53)		1.57 (1.04–2.38)	
Trimester of pregnancy				**0.08**		**0.13**
First	404	77	1			
Second	731	115	0.83 (0.64–1.07)		0.88 (0.68–1.14)	
Third	573	79	0.72 (0.54–0.96)		0.77 (0.58–1.02)	
Parity				< **0.001**^a^		**0.63**
Multigravida	1039	138	1		1	
Secundigravida	314	62	1.31 (1.00–1.73)		1.10 (0.80–1.49)	
Primigravida	354	71	1.70 (1.32–2.20)		1.21 (0.83–1.76)	
Insecticide‑treated nets				**0.99**		**0.88**
At least one	1650	262	1		1	
No insecticide-treated nets	57	9	0.99 (0.54–1.83)		0.89 (0.50–1.61)	
Education				**0.56**		**0.86**
No education	1693	268	1		1	
Educated	14	3	1.35 (0.49–3.71)		1.24 (0.46–3.33)	
Household socioeconomic				**0.90**		**0.85**
Middle quintile	352	52	1		1	
Poorest	300	51	1.15 (0.81–1.64)		1.10 (0.78–1.55)	
Poor	364	57	1.06 (0.75–1.49)		1.05 (0.75–1.48)	
Rich	338	51	1.02 (0.71–1.45)		1.05 (0.74–1.49)	
Richest	354	60	1.14 (0.81–1.61)		1.18 (0.84–1.65)	
Place of residence				**0.84**		**0.91**
Rural	1586	251	1		1	
Urban	120	20	1.05 (0.69–1.58)		0.96 (0.64–1.43)	
Malaria season				< **0.001**^a^		< **0.001**^a^
Low season	1222	155	1		1	
High season	485	116	1.89 (1.52–2.34)		1.85 (1.49–2.29)	

*PR* prevalence ratio, *Adj*. adjusted, *MiP* malaria in pregnancy, ^a^Significant at the 0.05 level

**Table 3:** Risk factors associated with asymptomatic carriage among pregnant women surveyed at the household level in Burkina Faso in 2017

Potential factors	N	MiP cases	Univariate analysis		Multivariable analysis	
			PR (95% CI)	p	Adj. PR (95% CI)	p
Age				< **0.001**^a^		< **0.001**^a^
≥ 30	623	18	1		1	
20–30	275	41	5.16 (3.02–8.82)		1.71 (1.01–2.91)	
≤ 20	933	58	2.15 (1.28–3.62)		2.73 (1.32–5.67)	
Trimester of pregnancy				< **0.001**^a^		< **0.001**^a^
First	426	21	1		1	
Second	709	53	0.74 (0.5–1.09)		0.71 (0.49–1.03)	
Third	697	43	0.30 (0.18–0.50)		0.28 (0.17–0.47)	
Parity				< **0.001**^a^		**0.052**
Multigravida	1325	55	1		1	
Secundigravida	310	32	2.49 (1.64–3.78)		1.75 (1.07–2.87)	
Primigravida	196	30	3.69 (2.43–5.61)		2.07 (1.10–3.90)	**0.10**
Insecticide-treated nets				**0.020**^a^		
At least one	1762	108	1		1	
No insecticide-treated nets	69	9	2.13 (1.13–4.02)		1.58 (0.80–3.11)	
Education				**0.58**		**0.81**
No education	1755	111	1		1	
Educated	76	6	1.25 (0.57–2.75)		1.09 (0.49–2.4)	
Household socioeconomic				**0.14**		**0.59**
Middle quintile	360	23	1		1	
Poorest	334	31	1.45 (0.87–2.44)		1.28 (0.76–2.15)	
Poor	363	22	0.95 (0.54–1.67)		0.93 (0.53–1.62)	
Rich	375	17	0.71 (0.39–1.31)		0.83 (0.46–1.52)	
Richest	399	24	0.94 (0.54–1.64)		0.92 (0.55–1.56)	
Place of residence				**0.10**		**0.15**
Rural	1685	103	1		1	
Urban	146	14	1.57 (0.92–2.67)		1.46 (0.87–2.44)	

*PR* prevalence ratio, *Adj.* adjusted, *MiP* malaria in pregnancy, ^a^Significant at the 0.05 level

### Factors associated with asymptomatic carriage among pregnant women surveyed at the household level

The results of multivariable analyses to identify potential factors associated with asymptomatic carriage are summarized in Tables 2 and 3 for 2013/2014 and 2017, respectively. The results show previously identified risk factors for MiP, namely young maternal age, primigravidae, the first trimester of pregnancy, and the high malaria transmission season. Regarding the transmission season, the prevalence of asymptomatic carriage of  malaria parasites was 1.83 (95% CI 1.47–2.29) times higher during the high transmission season compared to that of the low transmission season. During the year 2017, consisting only of women recruited during the low transmission season, i.e. between April 2017 and June, the prevalence of asymptomatic carriage halved and was estimated at 6.4% (95% CI 5.3–7.6). There was no statistical association between asymptomatic carriage of malaria parasites and ownership of insecticide-treated nets, household socioeconomic status, education, nor place of residence.

### Prevalence of anaemia among pregnant women surveyed at the household level

**Fig. 2:**
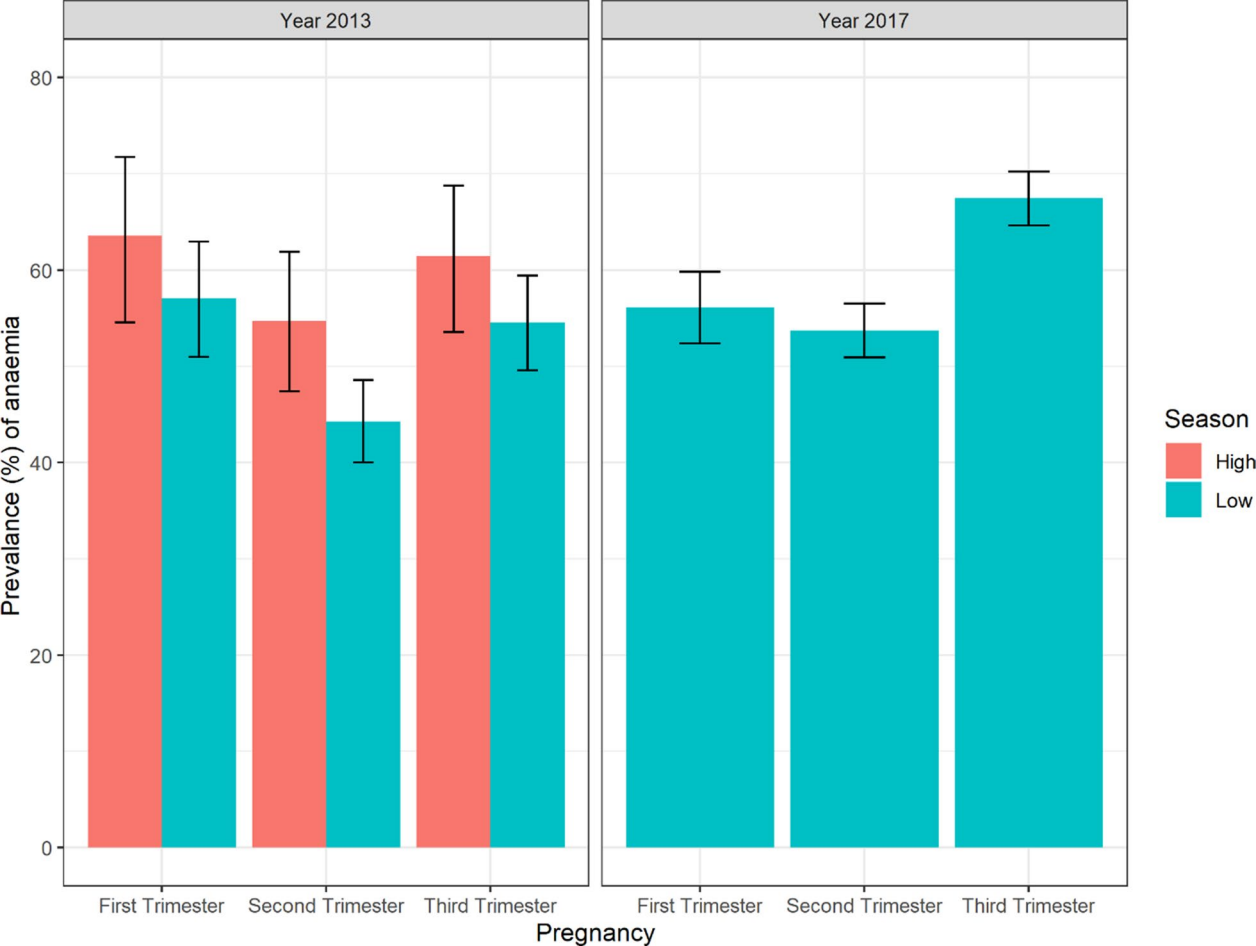
Prevalence of anaemia by trimester of pregnancy according to the malaria transmission season. The anaemia in pregnancy was defined as follow: Hb < 11 g/dL in the first trimester, < 10.5 g/dL in the second trimester, and < 11 g/dL in the third trimester. The high transmission season in 2013 covered the months between October and November. The low transmission season in 2013–2014 covered the months between December 2013 and March–2014. The low transmission season in 2017 covered the months between April and June 2017

In 2013/2014, the mean haemoglobin was estimated at 10.5 (SD = 1.6) g/dL and prevalence of anaemia was 35.9% (95% CI 33.6–38.2). In year 2017, the mean haemoglobin was 10.2 (SD = 1.4) and prevalence of anaemia was 46.6% (95% CI 44.3–48.9). The prevalence of anaemia 95% CIs by trimester of pregnancy and according to malaria transmission season is presented in the Fig. 2. During the low transmission season of 2014 (i.e., December to March), the prevalence of anaemia was estimated at 32.7% (95% CI 30.1–35.5). During the high transmission season of 2013 (i.e., October to November), the prevalence of anaemia was 43.7% (95% CI 39.3–48.3). During the high season the prevalence of anaemia was estimated at 59.4% (95% CI 50.7–67.6), 50.0% (95% CI 43.3–56.7), and 58.3% (95% CI 50.6–65.6), respectively at first, second and third trimester of pregnancy.

### Risk factors associated with anaemia among pregnant women surveyed at the household level

In general, as shown in Tables 4 and 5, the prevalence of anaemia was higher in pregnant women with malaria than in those who had no malaria infection. Likewise, the prevalence of anaemia was lower among pregnant women with the richest household socioeconomic status. After adjustment, we observed a lower prevalence of anaemia among women in the first trimester of pregnancy, which was more noticeable for the year 2017 (Table 5). Compared with non-infected pregnant women, asymptomatic carriage of malaria parasites was associated with a higher prevalence of anaemia (1.63; 95% CI 1.43–1.86). The results indicated that lower (poorest) socioeconomic status (Adj. PR: 1.20; 95% CI 1.03–1.39) and high transmission season (Adj. PR: 1.30; 95% CI 1.13–1.5) were significantly associated with anaemia. The prevalence of anaemia did not differ according to parity, ownership of insecticide-treated nets, education, nor place of residence.

**Table 4:** Potential risk factors of anemia among pregnant women surveyed at the household level in Burkina Faso in 2013/2014

Potential factors	N	Anemia cases	Univariate analysis		Multivariable analysis	
			PR (95% CI)	p	Adj. PR (95% CI)	p
Asymptomatic malaria				< **0.001**^a^		< **0.001**^a^
No	1436	479	1		1	
Yes	271	133	1.34 (1.22–1.48)		1.31 (1.18–1.44)	
Age				**0.21**		**0.42**
≥ 30	468	153	1		1	
20–30	859	305	1.07 (0.96–1.19)		1.10 (0.98–1.23)	
≤ 20	380	154	1.12 (0.99–1.27)		1.19 (1.00–1.41)	
Trimester of pregnancy				< **0.001**^a^		< **0.001**^a^
First	404	239	1		1	
Second	731	343	0.79 (0.71–0.89)		0.81 (0.72–0.90)	
Third	573	324	0.96 (0.86–1.07)		0.97 (0.87–1.08)	
Parity				**0.99**		**0.28**
Multigravida	1039	360	1		1	
Secundigravida	354	130	1.00 (0.89–1.12)		0.89 (0.76–1.05)	
Primigravida	314	122	1.00 (0.89–1.13)		0.92 (0.82–1.05)	
Insecticide-treated nets				**0.41**		**0.38**
At least one	1650	589	1		1	
No insecticide-treated nets	57	23	0.89 (0.67–1.17)		0.87 (0.66–1.15)	
Education				**0.56**		**0.48**
No education	1696	608	1		1	
Educated	11	4	1.21 (0.82–1.80)		1.19 (0.75–1.90)	
Household socioeconomic				**0.75**		**0.67**
Middle quintile	351	132	1		1	
Poorest	300	101	0.95 (0.82–1.10)		0.95 (0.82–1.09)	
Poor	364	143	1.03 (0.90–1.17)		1.04 (0.91–1.18)	
Rich	338	133	1.02 (0.89–1.17)		1.02 (0.89–1.17)	
Richest	354	103	0.95 (0.83–1.10)		0.95 (0.83–1.09)	
Place of residence				**0.27**		**0.27**
Rural	1586	568	1		1	
Urban	121	44	0.90 (0.74–1.09)		0.89 (0.73–1.08)	
Malaria season				**0.001**^a^		**0.008**^a^
Low season	1222	400	1		1	
High season	485	212	1.18 (1.07–1.29)		1.13 (1.03–1.24)	

*PR* prevalence ratio, *Adj.* adjusted,^a^ significant at the 0.05 level

**Table 5:** Potential risk factors of anaemia among pregnant women surveyed at the household level in Burkina Faso in 2017

Potential factors	N	Anaemia cases	Univariate analysis		Multivariable analysis	
			PR (95% CI)	p	Adj. PR (95% CI)	p
Asymptomatic malaria				< **0.001**^a^		< **0.001**^a^
No	1714	770	1		1	
Yes	117	83	1.37 (1.27–1.48)		1.47 (1.34–1.60)	
Age				**0.60**		**0.56**
≥ 30	623	290	1		1	
20–30	933	426	0.97 (0.90–1.05)		0.95 (0.88–1.03)	
≤ 20	275	137	1.02 (0.92–1.13)		0.93 (0.81–1.07)	
Trimester of pregnancy				< **0.001**^a^		< **0.001**^a^
First	426	236	1		1	
Second	709	432	1.10 (0.99–1.22)		1.11 (1.00–1.23)	
Third	697	523	1.35 (1.23–1.49)		1.39 (1.27–1.53)	
Parity				**0.64**		**0.94**
Multigravida	1325	608	1		1	
Secundigravida	310	140	1.05 (0.94–1.16)		1.02 (0.89–1.18)	
Primigravida	196	105	1.03 (0.94–1.12)		1.02 (0.93–1.13)	
Insecticide-treated nets				**0.39**		**0.54**
At least one	1762	817	1		1	
No insecticide-treated nets	69	36	1.07 (0.91–1.26)		1.02 (0.87–1.21)	
Education				**0.75**		**0.97**
No education	1755	819	1		1	
Educated	76	34	1.01 (0.86–1.26)		1.00 (0.85–1.18)	
Household socioeconomic				**0.022**^a^		**0.06**
Middle quintile	360	161	1		1	
Poorest	334	182	1.12 (1.01–1.23)		1.10 (0.99–1.21)	
Poor	363	169	0.97 (0.87–1.08)		0.97 (0.87–1.08)	
Rich	375	180	1.02 (0.92–1.13)		1.01 (0.91–1.13)	
Richest	399	161	0.95 (0.85–1.06)		0.96 (0.86–1.07)	
Place of residence				**0.43**		**0.32**
Rural	1685	789	1		1	
Urban	146	64	0.95 (0.83–1.08)		0.94 (0.83–1.06)	

*PR* prevalence ratio, *Adj.* adjusted, ^a^ significant at the 0.05 level

## Discussion

The presence of malaria and anaemia in pregnancy, regardless of the gestational stage, are potentially harmful to both the fetus and mother as well as to the family and community [1, 2, 5–8, 37, 38]. This study provides insights into the extent of asymptomatic MiP and its corollary, anaemia, at the community level (i.e., pregnant women surveyed in their own family home) throughout different malaria transmission periods of the year in Burkina Faso.

The study findings show that the prevalence of asymptomatic MiP and anaemia at the household level was high. In this present study, the MiP prevalence was higher among young women, increased significantly during the high transmission season and increased the risk of maternal anaemia. For the survey conducted in 2017, the risk of MiP was higher during the first trimester of pregnancy.

The prevalence of asymptomatic carriage of malaria parasites found in this household-based study was lower compared with the estimated prevalence reported in previous studies, where it was reported to range from 19 to 51%, among pregnant women attending health facilities as part of their ANC in Burkina Faso [25–28]. However, the overall aggregate prevalence of asymptomatic MiP (11.0%) reported in our study corroborates the results (12.2%) from the COSMIC household-based survey conducted in Burkina Faso between March 2014 and January 2016 [29, 39]. In addition, the prevalence of asymptomatic MiP at the community level during the low transmission season was slightly lower compared to the prevalence of 9.1% reported from a community-based study conducted in Ethiopia during the minor (“low”) malaria transmission season [40]. Therefore, the results underline the need for maintaining effective prevention measures throughout the entire course of pregnancy. These results showed that pregnant women living in Burkina Faso (or other SSA countries) are consistently exposed to malaria risk and its harmful consequences at any time of the year. However, the highest risk of asymptomatic carriage of malaria parasites in the study seemed to occur among younger women (primi- or secundigravida), in the second and third trimester of pregnancy, and during the high malaria transmission season*.* These risk factors have been documented in other previous studies [10, 25–27, 33, 34, 41–43]. Thus, more efforts are needed to increase IPTp–SP coverage (i.e. monthly from the second trimester to delivery), at both the health facility level and community/household level through the community-based health workers (CHWs), in order to reduce anaemia and contribute to preventing low birth weight prevent poor pregnancy outcomes [19, 44]. From this perspective and according to the WHO recommendations [45], several countries, including Burkina Faso, have set up strategies involving CHWs whose tasks include community-based sensitization activities, prevention measures, conducting malaria home diagnoses by RDT, and treatment of uncomplicated malaria within their respective communities [29, 46].

The study showed a high prevalence of anaemia among pregnant Burkinabe women living in their communities, though this prevalence seemed to be slightly lower compared with the prevalence among pregnant women attending health facilities as part of their ANC [27, 28]. According to the literature, the nutritional deficiencies, particularly deficiency in iron and folic acid constitute the main causes of anaemia in pregnancy. The difference in prevalence of anaemia observed during the transmission season (especially between October–November 2014 and April June 2017) could be also explained by the food shortage period in Burkina Faso. In addition to the nutritional factors, in our context, malaria (sequestration of parasitized red blood cells in the placenta), second trimester of pregnancy, and the high malaria transmission season may not be the only causes of anaemia in pregnant women. Indeed, the causes of anaemia during pregnancy in developing countries are multifactorial and may be a result of other co-morbidities (worm infestation), complication events (including placenta previa, placental abruption), chronic diseases (HIV, sickle cell disease, and TB), or nutritional deficiency (inadequate intake of iron and folic acid and/or inadequate iron + folic acid supplementation) [47–53]. Appropriate community-based strategies to prevent anaemia in pregnancy can help to significantly reduce the occurrence of maternal anaemia and, thereby, avoid progression to fatal outcomes. In this respect, the capacity of CHWs should be strengthened to allow them to carry out targeted sensitization of the risk factors leading to anaemia, ensure effective adherence to preventive measures among the community, detect clinical signs of anaemia (pallor, fatigue, bleeding), and direct people to a health centre for appropriate clinical management (such as administering of a double dose of iron) [54].

Although the findings of this study provide an overview of the extent of asymptomatic malaria and anaemia in pregnant women at the community level, both during the high and the low transmission seasons in Burkina Faso, some potential limitations need to be considered. First, asymptomatic carriage of malaria parasites was defined by using a malaria RDT, which could lead to underestimation (due to false negatives) or overestimation (false positives) of the true prevalence. False negatives from the malaria RDT may be due to the detection threshold of the test (around 200 parasites/μL) [55]. Regarding the false negative results, some studies found that more than half of all false negatives were in cases of parasitemia in which the detected antigen was under the detection threshold (i.e., lower density). False positives could be due to prolonged antigen circulation following clearance of malaria parasites. Indeed, it was shown that HRP2 antigens can persist in the bloodstream of pregnant women for up to four weeks after successful treatment [56]. However, in high-transmission areas such as Burkina Faso, HRP2 RDT could be a useful tool for malaria diagnosis, since previous studies found its performance was better than that of microscopy when used in pregnant women [31]. Second, the cross-sectional study design did not allow us to determine cause and effect. Third, the two survey periods (not overlapping) did not allow us to compare outcomes over time, although this was not in the scope of this study. Fourth, the estimation of the gestational age through mainly the recall of last menstrual cycle could introduce classification bias in the estimation of  trimester of pregnancy. The absence of indicator related to the use of ITNs by the pregnant women (a good proxy of protection conferred by bed nets) did not allow us to raise the effect of protection conferred by ITNs. In addition, information regarding the number of doses taken by pregnant woman was not available. However, since the IPTp-SP is planned to be systematically given to the pregnant women after the first trimester of pregnancy, regardless the month of the year, suggests that there was not difference related to season. Therefore, the absence of any benefit of bed nets ownership should be interpreted with caution.

## Conclusion

This study showed a high prevalence of both asymptomatic malaria and anaemia during pregnancy, and it indicated the risks increased dramatically during the high transmission season. Thus, infected anaemic (or non-anaemic) women, apart from consequences to their unborn babies (being considered as reservoirs of parasites that may promote mother-to-child transmission), represent an important parasite reservoir contributing to the cycle of malaria transmission in the community. In order to mitigate the harmful effects of asymptomatic carriage of malaria parasites and anaemia for both the mother and her fetus, health programmes aimed at increasing the number of pregnant women coming into contact with health workers should be at least maintained or strengthened. Therefore, more efforts are needed to increase IPTp–SP coverage, at both the health facility and community/household level, to prevent poor pregnancy outcomes [19, 44]. This could be implemented by strengthening the activities of community-based health workers (regular screening for malaria and anaemia in villages and households) in order to reduce the progression of asymptomatic cases to clinical, or even severe cases before pregnant women can reach the health centres for ANC.

## Data Availability

The dataset containing individual and household level records is available at the Centre MURAZ in Burkina Faso.

## Abbreviations

Adj.: Adjusted
ANC: Antenatal care
CAPI: Computer-assisted personal interview
CHW: Community-based health workers
CI: Confidence intervals
GEE: Generalized estimating equations
HIV: Human immunodeficiency viruses
HRPII: Histidine-rich protein II
IPTp: Intermittent preventive treatment during pregnancy
MiP: Malaria infection in pregnancy
PR: Prevalence ratios
RDT: Rapid diagnostic tests
SP: Sulfadoxine–pyrimethamine
SSA: Sub-Saharan African
TB: Tuberculosis
WHO: World Health Organization
